# Infertility

**DOI:** 10.1155/2012/508276

**Published:** 2012-03-11

**Authors:** Mittal Suneeta, Dhaliwal Lakhbir, Sharma Sanjeev, Chauhan Sanjay, B. S. Garg, Neeta Singh

**Affiliations:** ^1^Department of Obstetrics & Gynaecology, All India Institute of Medical Sciences, New Delhi 110029, India; ^2^Postgraduate Institute of Medical Education & Research, Chandigarh 160012, India; ^3^Southport and Ormskirk Hospital NHS Trust, Southport, Merseyside, UK; ^4^National Institute for Research in Reproductive Health, Mumbai 400074, India; ^5^Mahatma Gandhi Institute of Medical Sciences, Sevagram, Wardha, India

Infertility is acquiring a proportion of global epidemic with the prevalence rate of approximately 8–10% according to World Health Organization. Reproductive health implies individual's right to reproduce and freedom to decide when and how often to have children. The couples have a right to have children and right to access appropriate health care services that will enable them to achieve their goal. Infertility, however, continues to be a worldwide problem, affecting an estimated 60–80 million women and men worldwide, a vast majority of whom live in resource poor countries. 

The problem in low-resource countries is compounded because of several factors specially related to unhygienic obstetrics and postabortal practices. Most low-resource countries also have high population growths compared to the developed countries. Increasing population understandably dilutes the health care funding; however infertile couples are denied treatment for their suffering in the name of not increasing the already high population growth, a problem which the infertile couples did not contribute to.

Infertility may not be a threat to physical health but carries with it extremely adverse social and psychological implications for all concerned but particularly in developing countries. Since it is accepted that individuals cannot achieve health in general and reproductive health in particular without the alleviation of infertility there is an ever-increasing need and urgency to develop simple, low-cost, and effective instruments for evaluation, treatment, and prevention of infertility, which can be applied universally.

Infertility thankfully is not considered a “female” only problem; however lack of awareness still ensures that the women bear the brunt of the blame and its associated reactions. Current understanding of the pathophysiology of infertility based on the newer technologies, for example, structural genetics, molecular biology, and imaging techniques has significantly improved the management strategies. This understanding has also made us better aware of the well known pathologies however these need to be justified and applied carefully in low-resource countries making the interventions specific, clinically relevant, and cost-effective.

With idea of focused reading publication of special issues on specific important area is the unique quality of this journal. Infertility is an area that needs specific attention by dedicated physicians working in this specialty. Quality of submitted manuscripts and their review process has been very meticulous. The current issue has looked at the well-known pathologies and reviewed them in the light of the current understanding. This issue highlights the various topics dealing with social, epidemiological, causative, and treatment modalities and recent advances in management of infertility. 

Infertility is multifaceted condition with a myriad of causes and treatment. This has been possible due to tremendous research and improvement in the reproductive and genetic technologies. However, it is important not only to have a basic background of facts and fundamental principles, but also revisit and revise our perspectives of treatments for infertility. 

Ovulatory dysfunction appears to be on the rise with changes in life style, increase in stress and strain, late child bearing practices, and rising incidence of polycystic ovarian disease. Induction of ovulation remains backbone of infertility treatment, and clomiphene citrate has earned its fame as ovulation-inducing agents for almost half a century. With now availability of Letrozole (though not approved in many countries yet) ovulation results are as good as clomiphene; in addition women with thin endometrium get good response; the article on its use in polycystic ovary syndrome provides its dosage optimization which so far is not very clear as the dosage of clomiphene is standardized.

Assisted reproduction is one of the fastest growing areas of medicine having expanded far beyond the imaginations of those who pioneered the techniques that led to the birth of Louise Brown. Thirty-odd years after her birth, infertility treatment has improved substantially. Not only has the process of uniting egg and sperm outside the body become a commonly practiced procedure, assisted conception treatments are proving to be more efficient and cost-effective. The availability of cryopreservation technology has extended the scope of infertility treatment and proved to be boon for patients. Assisted hatching, preimplantation genetic diagnosis, ovarian tissue freezing, stem cell technology, gamete donation, embryo donation, and surrogacy, these treatment options are mind-boggling. This requires the healthcare provider to be well equipped with the present scenario. Review article by P. R. Brezina and Y. Zhao has done good justice to ethical, legal, and social issues impacted by modern ART.

Review articles on important chronic benign problem of endometriosis and adenomyosis are very informative. In the era of stem cell therapy the article on endometrial stem cells and reproduction should sensitize the readers to work and do more research in this area since a significant number of women with damaged endometrium would benefit from this mode of therapy especially in developing countries where endometrium is destroyed due to genital tuberculosis in addition to other causes. Studies are required to delineate the mechanisms responsible for successful treatment of Asherman's syndrome, an intractable disease. 

Genetic variation and environmental factors contribute susceptibility to spermatogenic impairment in human, the article on sperm DNA integrity assessment reviews use of this new tool in the diagnosis of male infertility. Every patient with tubal factor infertility cannot afford assisted reproduction, and good number of them may benefit from tubal reconstructive surgery, but the postoperative adhesions formation is a big hurdle to success the article on prevention of postoperative adhesions with viscous liquid explores very economical mode of preventive measures and makes an interesting reading. Many more community-based epidemiological studies on the infertility need to be done like the one from Pakistan.

In this special issue the topics have been arranged as epidemiology followed by the causative factors, investigations, infertility management, and its effect/side effects and include both original research and review articles.

*Mittal Suneeta* *Mittal Suneeta* *Dhaliwal Lakhbir* *Dhaliwal Lakhbir* *Sharma Sanjeev* *Sharma Sanjeev* *Chauhan Sanjay* *Chauhan Sanjay* *B. S. Garg* *B. S. Garg* *Neeta Singh* *Neeta Singh*

